# Evaluation of the composition and antimicrobial activities of essential oils from four species of Lamiaceae Martinov native to Iran

**DOI:** 10.1038/s41598-022-21509-5

**Published:** 2022-10-11

**Authors:** Mansureh Ghavam, Gianluigi Bacchetta, Ines Castangia, Maria Letizia Manca

**Affiliations:** 1 Department of Range and Watershed Management, Faculty of Natural Resources and Earth Sciences, University of Kashan, Kashan, Iran; 2 Department of Life and Environmental Sciences, University of Cagliari, Cagliari, Italy

**Keywords:** Biochemistry, Bioinorganic chemistry

## Abstract

In this study the essential oils obtained from four different plant species belonging to the Lamiaceae family were extracted by means of hydrodistillation and their composition and antimicrobial activity were evaluated. About 66 components were identified by using gas chromatography-mass spectrometry (GC–MS), and among all, thymol (67.7%), oleic acid (0.5–62.1%), (−)-caryophyllene oxide (0.4–24.8%), α-pinene (1.1–19.4%), 1,8-cineole (0.2–15.4%), palmitic acid (0.32–13.28%), ( +)spathulenol (11.16%), and germacrene D (0.3–10.3%) were the most abundant in all the species tested (i.e. *Thymus daenensis, Nepeta sessilifolia, Hymenocrater incanus*, and *Stachys inflata*). In particular, only the composition of essential oils from *H. incanus* was completely detected (99.13%), while that of the others was only partially detected. Oxygenated monoterpenes (75.57%) were the main compounds of essential oil from *T. daenensis*; sesquiterpenes hydrocarbons (26.88%) were the most abundant in *S. inflata*; oxygenated sesquiterpenes (41.22%) were mainly detected in *H. incanus* essential oil, while the essential oil from *N. sessilifolia* was mainly composed of non-terpene and fatty acids (77.18%). Due to their slightly different composition, also the antibacterial activity was affected by the essential oil tested. Indeed, the highest antibacterial and antifungal activities were obtained with the essential oil from *T. daenensis* by means of the inhibition halo (39 ± 1 and 25 ± 0 mm) against Gram-positive strains such as *Staphylococcus aureus* and *Aspergillus brasiliensis*. The minimal inhibitory concentration (MIC) and minimal bactericidal/fungicidal concentration (MBC/MFC) of the essential oils obtained from the four species varied from 16 to 2000 μg/mL and were strictly affected by the type of microorganism tested. As an example, the essential oils from *H. incanus* and *S. inflata* were the most effective against the Gram-negative bacterium *Pseudomonas aeruginosa* (MIC 16 and 63 μg/ml, respectively), which is considered one of the most resistant bacterial strain. Therefore, the essential oils obtained from the four species contained a suitable phytocomplexes with potential applications in different commercial area such as agriculture, food, pharmaceutical and cosmetic industries. Moreover, these essential oils can be considered a valuable natural alternative to some synthetic antibiotics, thanks to their ability to control the growth of different bacteria and fungi.

## Introduction

An increased interest in finding new and safe antimicrobial molecules from natural origin, especially from plants, has been detected in the last decades^1,2^. To this purpose the scientific community has focused its attention on natural, safe and effective antimicrobial molecules. In particular, essential oils have been traditionally used for their antimicrobial effects as topical or systemic drugs for bacterial and fungal infections, as preservative in food and topical ointments, as natural as biocide in ecological agriculture. The different molecules contained in the essential oils can exsert a synergistic effect provide a protection higher than that achieved by single molecules also reducing the multidrug resistance, which occurs in different infections and and inhibiting the contamination by foodborne pathogens^3–5^.

Essential oils obtained from Lamiaceae have gained considerable interest since their rich content in biological active molecules especially volatile molecules, such as monoterpenes, sesquiterpenes, and coumarins in some cases^5,6^. The antifungal and antimicrobial effect of several essential oils from this family, like thyme, peppermint, lavender, rosemary, peppermint, savory and marjoram, has been previous demonstrated^7,8^. The essential oil obtained from *Thymus daenensis* Čelak., endemic in NE Iraq and Iran, contains thymol, carvacrol, and p-cymene in high amount^9–15^. Thymol and β-caryophyllene, being the main components of *T. daenensis* essential oil, seem to be responsible of its antifungal and antibacterial effects^10,16–19^. As a function of its composition and origin, the essential oils of *T. daenensis* have been used to treat asthma, recurrent dry cough, and bronchitis as well as food ingredient^12,20^. The antimicrobial activity of some *Nepeta* species has been studied as well^21–23^. The essential oil from *Nepeta sessilifolia* Bunge, endemic in Iran and Pakistan, was especially effective against *Candida albicans*. β-caryophyllene and 1,8-cineole, α-pinene, β-pinene, trans-β-ocimene, germacrene-D, and caryophyllene oxide are the major constituents of the essential oil of *Hymenocrater incanus* Bunge an exclusive species of this genus in Iran^24^. Recent studies confirmed the potential of the secondary metabolites from this genus as antimicrobial antifungal, antiparasitic^25–27^. Germacrene-D, bicyclogermacrene, and α-pinene are the main components contained in the essential of *Stachys inflata* Benth., native from NE Turkey to Iran, which have antimicrobial effect, especially against *Staphylococcus aureus, Escherichia coli,* and *Pseudomonas aeruginosa*^28–32^.

The recent interest of consumers in food preservatives of natural origin has shifted the research interest towards the possible application of the essential oils to this propose. Several in vitro studies have established the efficacy of essential oils from Lamiaceae family taxa against common foodborne pathogens such as *Bacillus cereus*, *Escherichia coli* O157:H7, *Listeria monocytogenes*, *Salmonella* ser. *Enteritidis*, *Salmonella* ser. *Typhimurium* and *Staphylococcus aureus*.

Overall results disclosed that the cultivation region can strongly affect the composition and consequently the effectiveness of essential oils from Lamiaceae and their selectivity against different pathogens. Given that, it is important to simultaneously compare the efficacy of essential oils obtained from different species also testing different pathogens.

Accordingly, in this study, the essential oils from four different species of Lamiaceae were extracted and characterized and their efficacy was tested simultaneously using 12 strains of microorganisms.

## Materials and methods

### Plant material

Aerial parts of *T. daenensis*, *N. sessilifolia*, *H. incanus*, and *S. inflata*, were collected from the Daran region, located in Isfahan province of Iran (longitude: 46˚49ʹ02ʺ and latitude: 36˚54ʹ170ʺ). Permission for collection of plant materials obtained from the Agricultural Jahad Office and also the owner of the farm. The study is in compliance with relevant institutional, national, and international guidelines and legislation. The harvested specimens were transferred to the laboratory and exposed to free air in shade to dry. One sample of each whole plant was collected and pressed. The plant was identified and recorded at the herbarium of the University of Kashan. The plant was identified and recorded with code number 1018, 1019, 1020, 1021.

### Extraction of essential oils

After complete drying, the samples were grinded to obtain small particles and ensure the complete extraction of the bioactives; 100 g of each sample was subjected to extraction by means of hydrodistillation using a Clevenger apparatus for 5 h. The weight of essential oils collected after sodium sulphate dehydration was calculated accurately and essential oils was stored in closed bottles at 4 °C in the dark until further use^59,60^.

### Gas chromatography–mass spectrometry (GC–MS) analysis

The determination of the constituents of essential oil samples has been performed by means of GC–MS method. A chromatograph (Model 6890) Coupled with an N-5973 mass spectrometer made in USA and a HP-5MS Capillary Column with 5% methylphenylsiloxane static phase (Length 30 m, Internal Diameter 0.25 mm, Layer Static Thickness 0.25 μm) and ionization energy of 70 eV has been used for qualitative identification of the components. A temperature gradient has been used for the analysis, starting from 60 °C and then increasing the temperature (at a rate of 3 °C/min) up to 246 °C. The injector and detector temperature were set at 250 °C, the injection volume was 1 µl with 1.50 split and the helium carrier gas delivered at a flow rate of 1.5 ml/min. The chemical components of the essential oils were identified as a function of the retention indices about standards of n-alkane mixtures (C8–C20) and mass spectral data of each peak using a computer library (Wiley-14 and NIST-14 Mass Spectral Library) and comparing these data with those reported in the literature^33^.

### Antimicrobial activity

#### Tested microorganisms

Twelve microorganisms were used to evaluate the antimicrobial activity of the selected essential oils. Microbial strains were provided by the Iranian Research Organization for Science and Technology (IROST, CITY, Iran). *Staphylococcus epidermidis* (ATCC 12228), *S. aureus* (ATCC 29737) and *Bacillus subtilis* (ATCC 6633) were chosen as Gram-positive bacteria, while *Klebsiella pneumonia* (ATCC 10031), *Shigella dysenteriae* (PTCC 1188), *Pseudomonas aeruginosa* (ATCC 27853), *Salmonella paratyphi-*A serotype (ATCC 5702), *Proteus vulgaris* (PTCC 1182) and *Escherichia coli* (ATCC 10536) were the Gram-negative bacteria selected. *Aspergillus niger* (ATCC 16404), *Aspergillus brasiliensis* (PTCC 5011) and *Candida albicans* (ATCC 10231) were the fungal strains tested.

#### Agar diffusion method

Well plates 6.0 mm in diameter containing Müller Hinton agar were prepared, and 100 µL of bacterial suspensions with a half-McFarland turbidity equivalent in culture medium were cultured. The essential oil (30 mg/mL) was dissolved in dimethylsulfoxide (DMSO) and 10 μL (equivalent to 300 μg) of each essential oil was poured into the wells. The plates were incubated at 37 °C for 24 h and their antimicrobial activity was measured for each microorganism measuring, by the antibiogram ruler (in millimetres), the diameter of inhibition halos. Three replicates were performed for each strain and results were calculated as mean ± standard deviation. DMSO was used as negative control and gentamicin and rifampin for bacteria, and nystatin for fungi, were used to compare their antimicrobial power with those of the essential oils^61^.

#### MIC evaluation

The minimum concentration capable of inhibiting the growth of the bacteria or fungi (MIC) was calculated by microdilution method. The essential oils were dissolved in a mixture of TSB medium and DMSO at an initial concentration of 2000 μg/mL. The stock solutions were diluted to reach the following concentrations: 1000, 500, 250, 125, 62.5, 31.25 and 15.63 µg/mL. Experiments were performed by using 96-well microplates. 95 µL of culture medium, 5 µl of bacterial suspension with 0.5 McFarland dilution, and 100 µL of essential oil dilutions were added to each well, and then the plate was incubated at 37 °C for 24 h for bacterial strains and 48 h and 72 h at 30 °C for yeast^61^. The MIC was determined by the improvement of opacity or the change in colour as the lowest concentration that inhibited the visible growth (absence of turbidity).

#### Determination of minimum bactericide/fungicidal concentration (MBC/MFC)

To determine the minimum concentration capable of killing the bacterial or fungal strains, the same microdilution method was used. After 24 h of incubation of bacteria with the essential oils at different concentrations, 5 µL of the content of each well were inoculated with neutrin agar medium and incubated at 37 °C for 24 h for bacterial strains and 48 h and 72 h at 30 °C for yeasts. After incubation, the colony-forming units (CFUs) were enumerated^61^. The MBC/MFC was the lowest concentration able to effectively reduce the growth of microorganisms (99.5%).

### Statistical analysis of data

Results are expressed as the mean ± standard deviation. Analysis of variance (ANOVA) was used for multiple comparisons of means, and the Tukey’s test and Student’s *t*-test were performed to substantiate differences between groups using XL Statistics for Windows. The differences were considered statistically significant for p < 0.05.

## Results

### Composition of essential oils

The different essential oils were obtained from the four different Lamiaceae species from Iran. Their colour varied from pale yellow to dark yellow and the yield was 1.88% from *T. daenensis*, 0.2% from *N. sessilifolia*, 0.02% from *H. incanus,* and 0.14% from *S. inflata.*

### GC–MS analysis

The chemical composition of the essential oils was investigated using a GC–MS. About 51 components were identified. The composition of essential oils from *H. incanus* was completely detected (99.13%), while that of *T. daenensis* (97.44%), *S. inflata* (95.77%), and *N. sessilifolia* (84.56%), was only partially detected (Table 1). The main compounds of essential oil from *T. daenensis* were oxygenated monoterpenes (75.57%), from *S. inflata* were sesquiterpenes hydrocarbons (26.88%) and from *H. incanus* were oxygenated sesquiterpenes (41.22%). Differently, the essential oil from *N. sessilifolia* was mainly composed of non-terpene and fatty acids (77.18%).

**Table 1:** Main components and retention indice (RI) detected in the essential oils from *Thymus daenensis* (TD), *Nepeta sessilifolia* (NS), *Hymenocrater incanus* (HI), and *Stachys inflata* (SI)*.*

No	Compound (%)	RI	TD	NS	HI	SI	Molecular formula
1	α-Thujene	864	0.85	0.39	–	–	C_10_H_16_
2	α-pinene	871	1.09	–	–	1.52	C_10_H_16_
3	Camphene	888	0.82	–	–	0.55	C_10_H_16_
4	Sabinene	908	–	0.33	–	–	C_10_H_16_
5	β -pinene	912	0.43	–	–	–	C_10_H_16_
6	β -Myrcene	921	1.50	–	–	–	C_10_H_16_
7	α-Phellandrene	933	0.28	–	–	–	C_10_H_16_
8	α-Terpinene	943	1.71	–	–	–	C_10_H_16_
9	p-Cymene	953	5.16	–	–	–	C_10_H_14_
10	1,8-Cineole	957	3.52	0.91	0.22	1.65	C_10_H_18_O
11	γ-Terpinene	980	6.20	–	–	–	C_10_H_16_
12	α-Terpinolene	1002	0.26	–	–	–	C_10_H_16_
13	Linalool	1013	0.67	1.45	–	1.20	C_10_H_18_O
14	Camphor	1044		–	–	1.67	C_10_H_16_O
15	Borneol	1064	3.67	–		1.17	C_10_H_18_O
16	α-Terpineol	1080	–	0.50		0.91	C_10_H_18_O
17	Verbenone	1093				2.20	C_10_H_14_O
18	Thymol	1170	67.71				C_10_H_14_O
19	Acetic acid, bornyl ester	1129				1.29	C_12_H_20_O_2_
20	α-Copaene	1191				0.69	C_15_H_24_
21	β- Bourbonene	1197	–	1.03		–	C_15_H_24_
22	β-Elemene	1201		–	–	1.12	C_15_H_24_
23	trans-Caryophyllene	1224	2.99	0.67	3.68	–	C_15_H_24_
24	( +)-Aromadendrene	1232	0.15	–	–	–	C_15_H_24_
25	α-Humulene	1239	–	–	1.50	–	C_15_H_24_
26	Alloaromadendrene	1242		–	0.29	–	C_15_H_24_
27	α-Amorphene	1251		–	–	0.92	C_15_H_24_
28	β-Cubebene	1254	–	0.26	–	–	C_15_H_24_
29	Germacrene D	1255	–	–	0.30	10.26	C_15_H_24_
30	Bicyclogermacrene	1263	–	–	0.57	9.19	C_15_H_24_
31	β -Bisabolene	1268	0.50	–	–	–	C_15_H_24_
32	δ-cadinene	1277	–	0.34	–	4.7	C_15_H_24_
33	cis-α-Bisabolene	1286	0.40	–	–	–	C_15_H_24_
34	Elemol	1296	–	0.24	–	–	C_15_H_26_O
43	( +) spathulenol	1314		–	–	11.16	C_15_H_24_O
35	(−)-Caryophyllene oxide	1315	0.41	1.26	24.81	–	C_15_H_24_O
36	Viridiflorol	1321		–	–	2.26	C_15_H_26_O
37	(−)-Humulene epoxide II	1330	–	–	3.69	–	C_15_H_24_O
38	α-Chamigrene	1347	–		2.37	–	C_15_H_24_
39	α-Cadinol	1356	–		7.66	3.25	C_15_H_26_O
40	Caryophyllenol-II	1367	–		5.06	–	C_15_H_24_O
41	(3*S*,4*R*,5*S*,6*R*,7*S*)-aristol-9-en-3-ol	1374			–	1.51	C_15_H_24_O
42	Myristic acid	1414	–		1.31	–	C_14_H_28_O_2_
43	2-Pentadecanone, 6,10,14-trimethyl-	1445	–		1.39	0.89	C_18_H_36_O
44	Phthalic acid	1462	–		0.55	–	C_8_H_6_O_4_
45	Tridecane	1478	–		0.42	0.45	C_13_H_28_
46	Hexadecanoic acid = palmitic acid	1515	–		13.28	12.10	C_16_H_32_O_2_
47	Phytol	1580	–		9.22	2.14	C_20_H_40_O
48	Oleic acid	1600	0.49	62.09	23.53	20.75	C_18_H_34_O_2_
49	Stearic acid	1627	–	8.16	–	3.89	C_18_H_36_O_2_
50	Linoleic acid	1633	–	6.06	–	–	C_18_H_32_O_2_
51	Lauric acid	1692	–	0.87	–	–	C_12_H_24_O_2_
	Total		95.77	84.56	99.13	97.44	
	Monoterpenes hydrocarbons		18.26	0.72	0	2.07	
	Oxygenated monoterpenes		75.57	2.86	0.22	8.8	
	Sesquiterpenes hydrocarbons		4.04	2.3	8.71	26.88	
	Oxygenated sesquiterpenes		0.41	1.5	41.22	18.18	
	Others		0.49	77.18	48.98	41.55	

**Figure 1:**
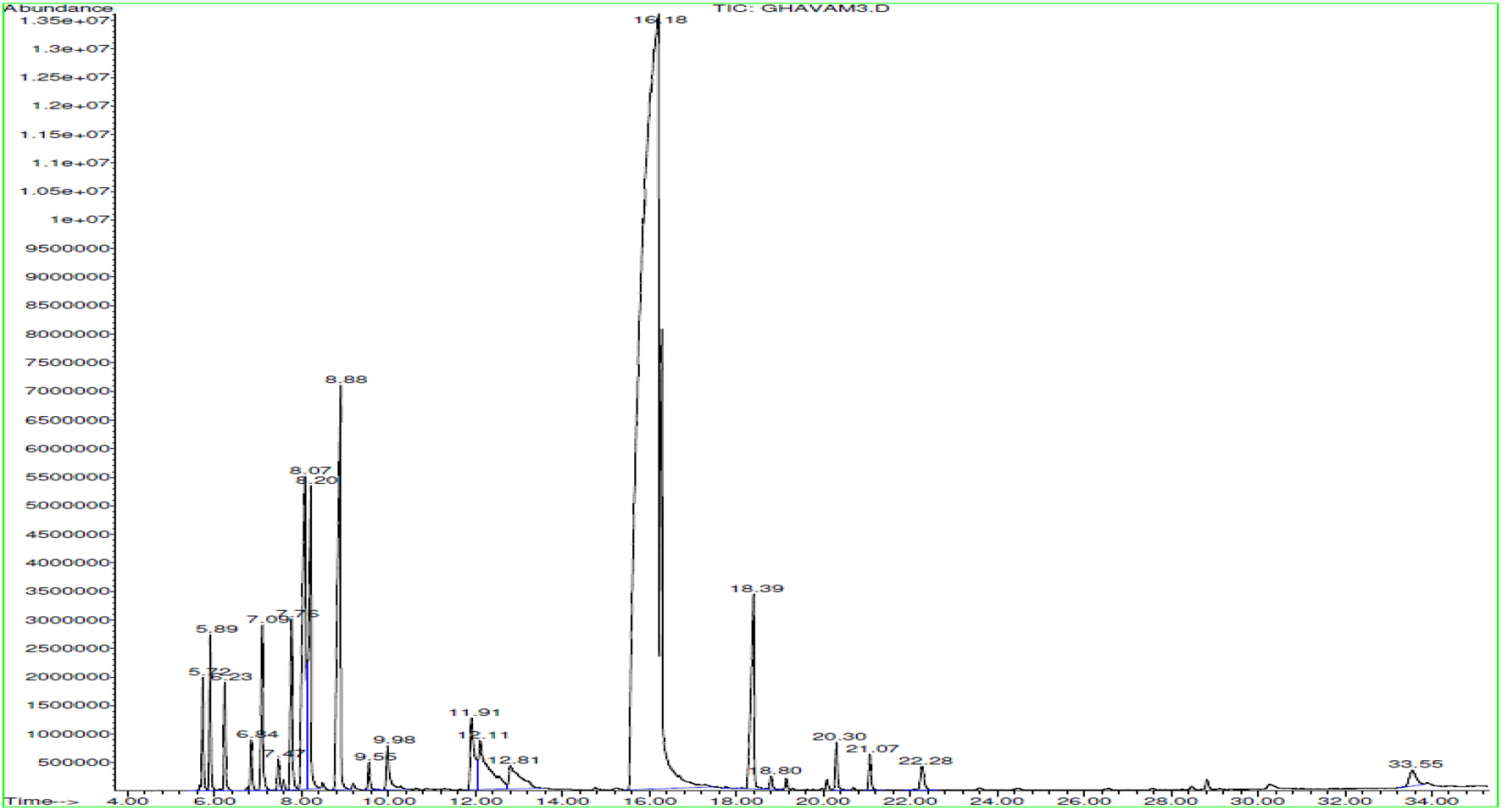
GC–MS chromatogram of essential oil obtained from *Thymus daenensis.*

The essential oil from *T. daenensis* was also rich in monoterpenes hydrocarbons (18.3%), and its main components were thymol (67.71%), γ-terpinene (6.20%), p-cymene (5.16%) and borneol (3.67%), and 1,8-cineole (15%) (Table 1 and Fig. 1).

**Figure 2:**
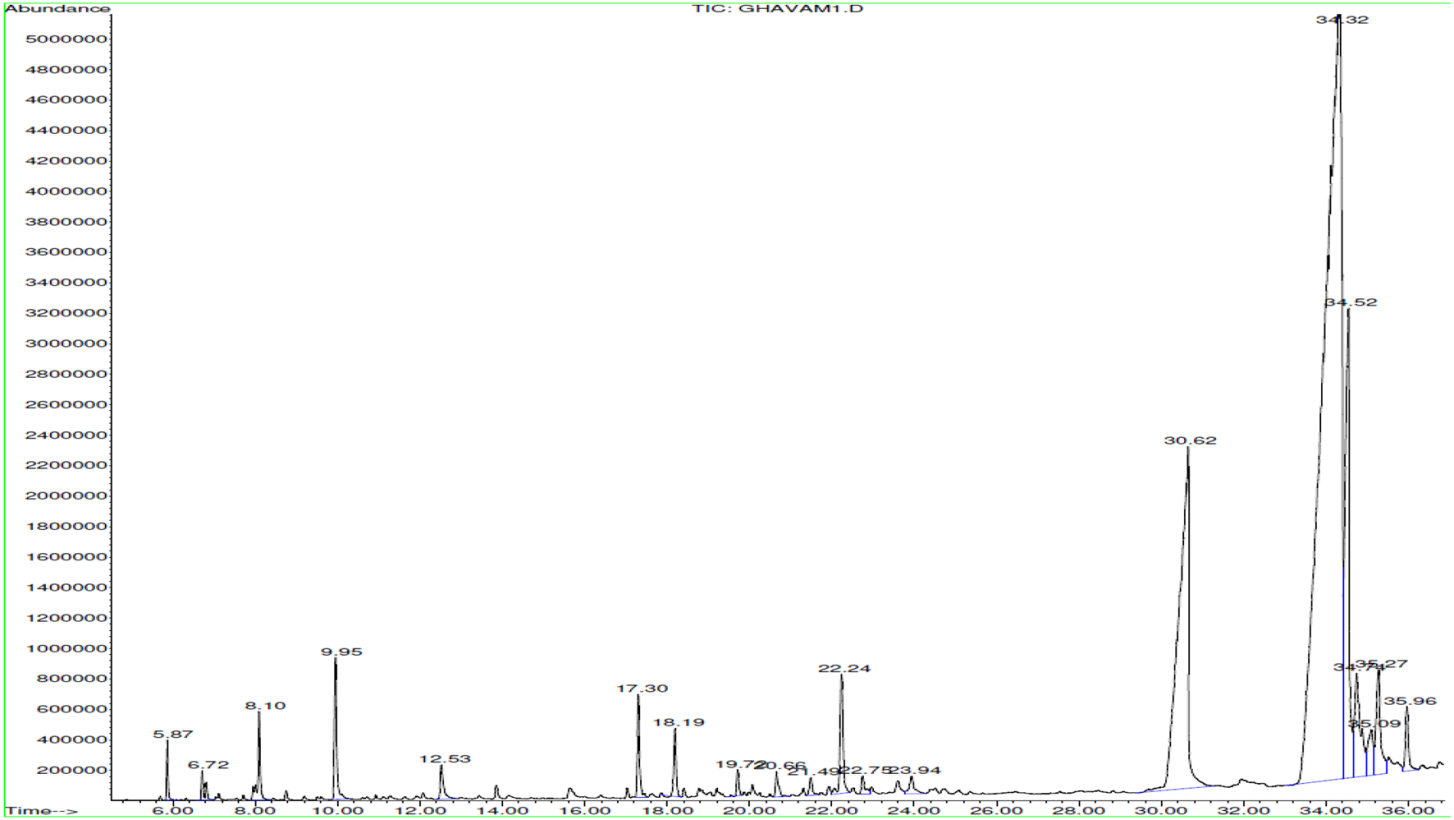
GC–MS chromatogram of essential oil obtained from *Nepeta sessilifolia.*

Fatty acids and non-terpenes are the main components of essential oil from *N. sessilifolia* (Table 1 and Fig. 2). In particular, oleic acid (62.09%), stearic acid (8.16%) and linoleic acid (6.06%) were detected for the first time in this oil.

**Figure 3:**
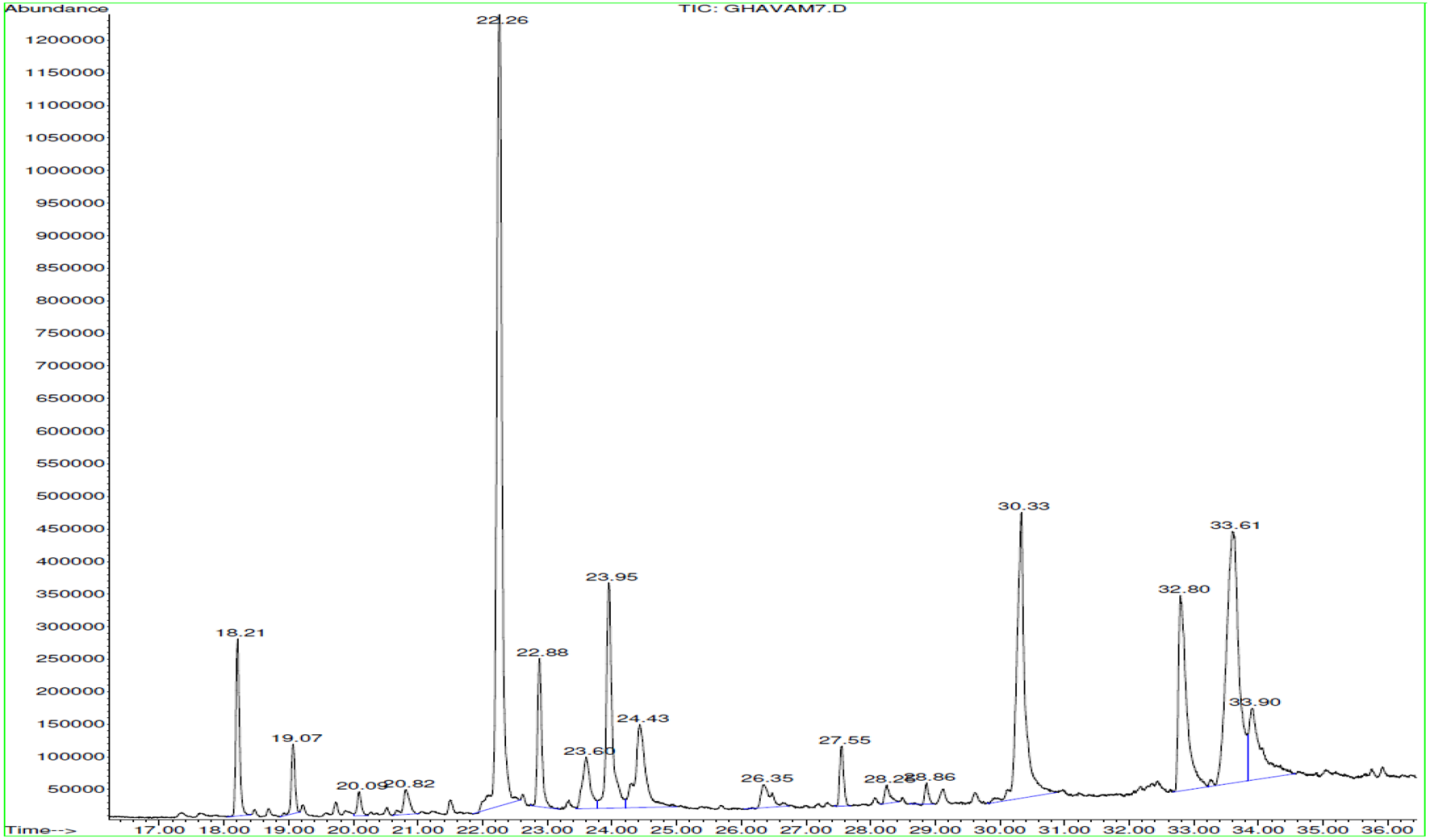
GC–MS chromatogram of essential oil obtained from *Hymenocrater incanus.*

Essential oil obtained from *H. incanus* was rich in fatty acids (50.65%) and oxygenated sesquiterpenes (41.22%). In particular, (−)-caryophyllene oxide (24.81%), oleic acid (23.53%), palmitic acid (28.23%), phytol (9.22%), α-cadinol (7.66%), and caryophyllenol-II were found (5.06%) (Table 1 and Fig. 3).

**Figure 4:**
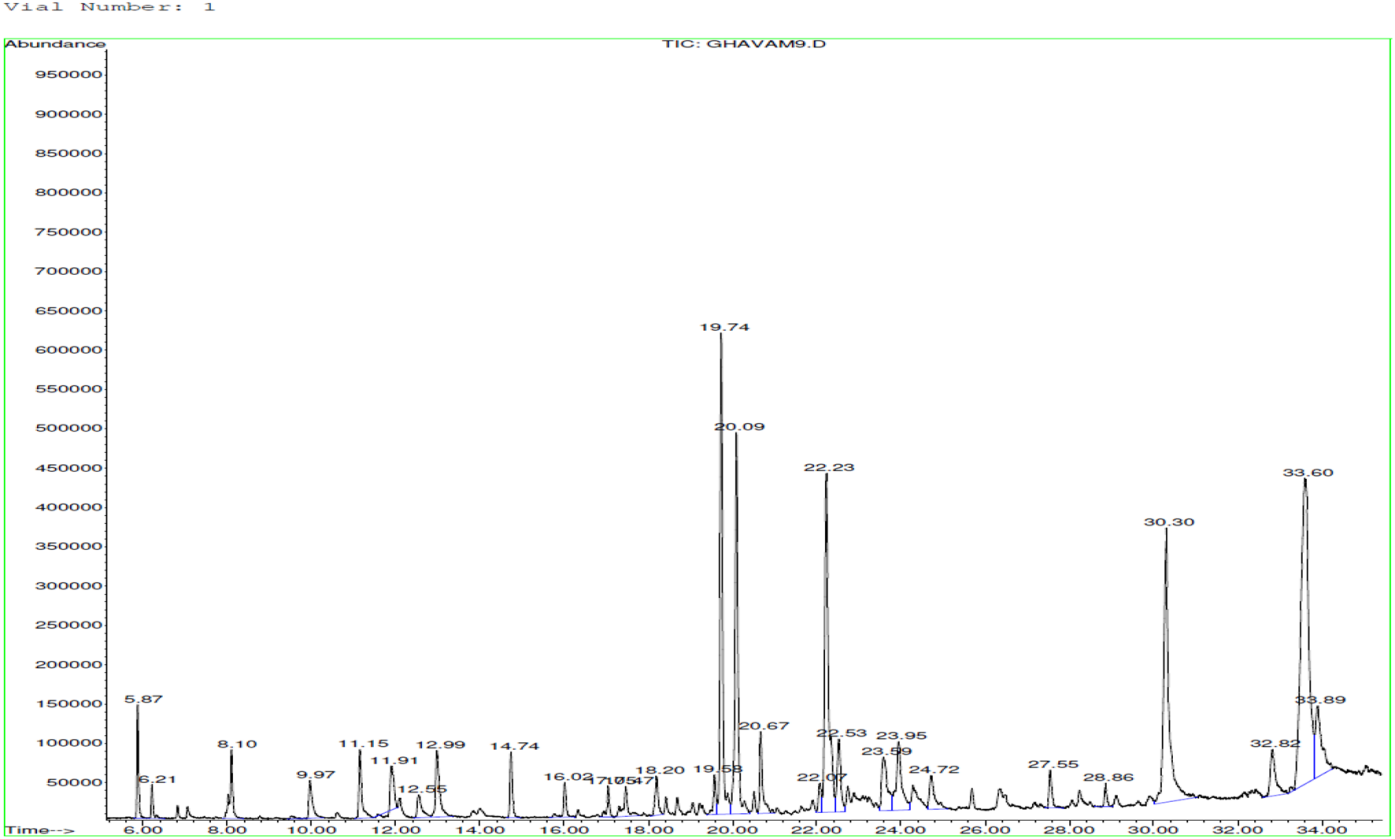
GC–MS chromatogram of essential oil obtained from *Stachys inflata.*

Acids (42.22%) and sesquiterpene hydrocarbons (26.25%), were the major constituents of the essential oil from *S. inflata*. Especially, oleic acid (20.75%), palmitic acid (12.12%), ( +)spathulenol (11.16%), germacrene D (12.26%), and bicyclogermacrene (9.9%) were detected (Table 1 and Fig. 4).

Overall data underlined that 1,8-cineole and oleic acid were commonly present in the essential oils of *T. daenensis*, *N. sessilifolia*, *H. incanus,* and *S. inflata.* The highest amount of 1,8-cineole was found in the oil from *T. daenensis* (3.52%) and the lowest in essential oil from *H. incanus* (0.22%). The highest amount of oleic acid was detected in the oil from *N. sessilifolia* (62.09%) while the lowest in that from *T. daenensis* (0.49%). Linalool was found in all essential oils except in that from *H. incanus*. Trans-Caryophyllene and (−)-Caryophyllene oxide were found in all essential oils except in that from *S. inflata* and the highest amount (3.68% and 24.81%) was found in oil from *H. incanus*. Thymol (67.71) and *p*-cymene (5.16) were contained only in the oil from *T. daenensis*; linoleic acid (6.06) and *S. inflata*; caryophyllenol-II (5.06) in the oil from *H. incanus*, ( +) spathulenol (11.16) and in the oil from *S. inflata*.

### Antimicrobial activity

#### Inhibition halos

The antibacterial and antifungal activities of the essential oils were measured to evaluate their potential applications (Table 2). The highest inhibition halo diameter (39 ± 1 mm) was obtained treating *S. aureus* with essential oil from *T. daenensis*, and it was even higher than that obtained by using rifampin (21 ± 0 mm) and gentamicin (27 ± 0 mm). Essential oil from *N. sessilifolia* (10 ± 1 mm) and *S. inflata* (9 ± 1 mm) had a significantly lower activity against this bacterium, while oil from *H. incanus* did not show any activity.

**Table 2:** Inhibition-zone diameters provided by antibiotics (used as references) and the essential oils from *Thymus daenensis* (TD), *Nepeta sessilifolia* (NS), *Hymenocrater incanus* (HI), and *Stachys inflata* (SI)*.*

Test microorganisms	IZ (mm)						
	Essential oils				Antibiotics		
	TD	NS	HI	SI	Rifampin	Gentamicin	Nystatin
*Sh. dysenteriae*	*16	ND	ND	ND	9	17	NA
*P. aeruginosa*	ND	ND	ND	ND	ND	20 .00	NA
*B. subtilis*	*^♪^ 14	*^♪^9	*^♪^10	*^♪^9	19	30	NA
*S. epidermidis*	*^♪^9	*^♪^9	*^♪^11	ND	44	39	NA
*E. coli*	^♪^11	ND	ND	ND	10	23	NA
*S. aureus*	*^♪^39	*^♪^10	ND	*^♪^9	21	27	NA
*K. pneumonia*	*18	ND	ND	ND	8	17	NA
*P. vulgaris*	*^♪^14	ND	ND	ND	8	24	NA
*S. paratyphi-A*	*^♪^12	ND	ND	ND	8	18	NA
*C. albicans*	^Ω^ 12	ND	ND	ND	NA	NA	33
*A. sniger*	^Ω^ 12	ND	ND	ND	NA	NA	27
*A. brasiliensis*	^Ω^ 25	ND	ND	ND	NA	NA	30

Mean values ± standard deviations of three cultures were reported.

*NA* no activity, *ND* not determined.

Symbols (*) indicate values statistically different from rifampin, symbols (♪) indicate values statistically different from gentamicin and symbols (Ω) indicate values statistically different from nystatin (p < 0.05).

Essential oil from *T. daenensis* was also effective against *A. brasiliensis* (25 ± 0 mm), and its activity was similar to that of nystatin (30 ± 0 mm), used as control. Moreover, oil from *T. daenensis* was capable of inhibiting the growth of *A. sniger* (12 ± 0 mm) and *C. albicans* (12 ± 1 mm) but in a lesser extent than nystatin (27 ± 0 and 33 ± 0 mm).

Essential oil from *T. daenensis* was also effective in counteracting two Gram-negative bacteria *K. pneumonia* (18 ± 1 mm) mm and *Sh. dysenteriae* (16 ± 0 mm) as the inhibition halos were larger than those obtained treating the same bacteria with rifampin (8 ± 0 mm and 9 ± 0 mm) and gentamicin (17 ± 0 mm). The oil was also effective against two more Gram-negative bacteria *E. coli* and *S. paratyphi-A*, being the inhibition halo 11 ± 1 and 12 ± 1 mm, which was slightly larger than that obtained by using rifampin (10 ± 0 and 8 ± 0 mm) and lower than that exerted by gentamicin (23 ± 0 and 18 ± 0 mm). It was also slightly effective against the Gram-positive *S. epidermidis* (9 ± 0), but any effect was detected against *S. paratyphi-A*, *S. epidermidis,* and *P. aeruginosa*.

Essential oil from *N. sessilifolia* was less effective than the essential oil obtained from *T. daenensis* as it inhibited the growth of only three Gram-positive bacteria: *B. subtilis* (14 ± 1 mm), *S. epidermidis* (9 ± 0 mm), and *S. aureus* (10 ± 1 mm). The efficacy of this oil was also lower than that obtained by using rifampin and gentamicin.

Essential oil from *H. incanus* inhibited only *B. subtilis* (10 ± 0) and *S. epidermidis* (11 ± 0 mm) in the less extend than rifampin and gentamicin. Essential oil from *S. inflata* had a weak activity against Gram-positive bacteria like *B. subtilis* (9 ± 0 mm) and *S. aureus* (9 ± 1 mm).

#### Measurament of MIC and MBC/MFC

The MIC of the essential oils varied from > 16 μg/mL to > 2000 μg/mL as a function of the microorganism and oil used (Table 3). The MIC (16 μg/mL) obtained treating the Gram-negative *P. aeruginosa* with essential oil from *S. inflata* were lower than that obtained using rifampin (31 μg/mL), but it was significantly higher than that obtained using gentamicin (8 µg/mL). The other essential oils had higher MIC (125 µg/mL) against this bacterium. The MIC of all the used oils (except that from *T. daenensis*) against *A. brasiliensis* and *A. niger* were around 2000 µg/mL, disclosing their inactivity and essential oil from *S. inflata* was not effective also against *S. aureus* (MIC > 1000 μg/mL).

**Table 3:** MIC obtained using antibiotics (used as references) and the essential oils from *Thymus daenensis *(TD), *Nepeta sessilifolia *(NS), *Hymenocrater incanus *(HI), and *Stachys inflata *(SI)*.*

Microorganisms	MIC (μg/mL)						
	Essential oils				Antibiotics		
	TD	NS	HI	SI	Rifampin	Gentamicin	Nystatin
*Sh. dysenteriae*	*^♪^ 500	*^♪^ 125	*^♪^ 63	*^♪^ 125	16	4	NA
*P. aeruginosa*	*^♪^ 125	*^♪^ 125	*^♪^ 16	*^♪^ 16	31	8	NA
*B. subtilis*	*^♪^ 125	*^♪^ 250	*^♪^ 500	*^♪^ 125	31	4	NA
*S. epidermidis*	*^♪^ 125	*^♪^ 250	*^♪^ 500	*^♪^ 500	2	2	NA
*E. coli*	*^♪^ 125	*^♪^ 500	*^♪^ 250	*^♪^ 125	16	31	NA
*S. aureus*	*^♪^ 125	*^♪^ 500	*^♪^ 500	*^♪^ > 1000	31	2	NA
*K. pneumonia*	*^♪^ 125	*^♪^ 125	*^♪^ 63	*^♪^ 125	16	4	NA
*P. vulgaris*	*^♪^ 250	*^♪^ 250	*^♪^ 500	*^♪^ 250	16	16	NA
*S. paratyphi-A*	*^♪^ 125	*^♪^ 250	*^♪^ 63	*^♪^ 125	16	4	NA
*C. albicans*	^Ω^ 31	^Ω^ 250	^Ω^ 63	^Ω^ 500	NA	NA	125
*A. sniger*	^Ω^ 250	^Ω^ 2000	^Ω^ > 2000	^Ω^ > 2000	NA	NA	31
*A. brasiliensis*	^Ω^ 250 ^b^	^Ω^ 2000 ^a^	^Ω^ > 2000	^Ω^ > 2000	NA	NA	31

Symbols (*) indicate values statistically different from rifampin, symbols (♪) indicate values statistically different from gentamicin and symbols (Ω) indicate values statistically different from nystatin (p < 0.05).

The MIC obtained treating the different bacteria with the oil from *T. daenensis* varied between 125 and 500 μg/mL, with its weakest inhibitory effect against *Sh. dysenteriae*. The antifungal activity of oil from *T. daenensis* was slightly higher (MIC from 31 to 250 μg/mL) and it especially inhibited *C. albicans* as its power was three times higher than that of nystatin (125 μg/mL).

The MBCs/MFCs obtained treating the different bacterial and fungal strains with oil from *T. daenensis* varied from 63 to 500 µg/mL (Table 4), the obtained MBCs/MFCs, irrespective of the microorganism tested, except *S. aureus*, *K. pneumonia* and *C. albicans*, were equal to the MICs (Table 3) indicating the capability of this oil of inhibiting the growth and killing the bacteria at the same concentration.

**Table 4:** MBC obtained using the essential oils from *Thymus daenensis *(TD), *Nepeta sessilifolia (*NS), *Hymenocrater incanus *(HI), and *Stachys inflata *(SI)*.*

Test microorganisms	MBC (μg/mL)			
	Essential oils			
	TD	NS	HI	SI
*Sh. Dysenteriae*	500	1000	*63	1000
*P. aeruginosa*	125	125	*16	250
*B. subtilis*	*125	> 1000	500	250
*S. epidermidis*	*125	250	500	500
*E. coli*	*125	500	250	500
*S. aureus*	500	1000	500	> 1000
*K. pneumonia*	250	125	*63	1000
*P. vulgaris*	*250	500	500	500
*S. paratyphi-A*	125	250	*63	500
*C. albicans*	*63	250	250	1000
*A. sniger*	*250	2000	> 2000	> 2000
*A. brasiliensis*	*250	2000	> 2000	> 2000

Symbols (*) indicate values statistically different from the others essential oils against the same bacterial/fungal strain (p < 0.05).

The MICs obtained by treating all microorganisms with essential oil from *N. sessilifolia* varied between 125 and 2000 μg/mL. The strongest effect was obtained against the Gram-negative *Sh. dysenteriae*, *K. pneumonia* and *P. aeruginosa*, but the found MICs were four times weaker than that provided by the antibiotics used as control. The lowest effect of this oil was against *A. niger* and *A. brasiliensis*. As reported for oil from *T. daenensis*, MBCs obtained treating the bacteria with oil from *N. sessilifolia* were always equal to MICs for all microorganisms tested (except *S. aureus*, *B. subtilis*, *Sh. dysenteriae*, and *P. vulgaris*). The lowest MBC of the essential oil from *N. sessilifolia* was obtained against *Sh. dysenteriae* and *K. pneumonia*, confirming its ability to inhibit and kill them. The low efficacy and high MICs and MBCs of essential oil from *N. sessilifolia* against most of the bacterial strains tested in this work, should be connected with the absence of terpenes (which are considered the most effective against bacteria). The antimicrobial power against few bacterial strains should be related to the fatty acid content, even if the mechanism of action of these compounds is still completely unknown and they are supposed to modify the permeability of the membrane and promote its disruption, thus causing significant alterations on the membrane-dependent conduction systems.

The MIC values obtained treating the different microorganisms with oil from *H. incanus* varied between > 16 and > 2000 µg/mL. The strongest activity was found against the Gram-negative *P. aeruginosa* (MIC 63 μg/mL)*.* The MIC of this oil against *C. albicans* was (63 µg/mL) lower than that of nystatin (MIC 125 µg/mL) while the MFC was significantly higher (250 µg/mL). Treating the other microorganisms, MBCs/MFCs were always equal to MICs and oxygenated sesquiterpenes such as (−)-caryophyllene oxide and α-cadinol contained can be responsible of these activities.

The MICs essential oil from *S. inflata* against the tested microorganisms varied between > 16 and > 2000 μg/mL. The strongest effect was found against the Gram-negative *P. aeruginosa,* being the MIC very low (16 μg/mL).

## Discussion

In previous studies, the yield of essential oil collected from *T. daenensis* was 2.09%^15^, from *N. sessilifolia* was 0.56%^34^, from *H. incanus* was 0.6%^24^, and from *S. inflata* was 2.9%^31^. Then the yields were always higher probably because the used plants were grown in different areas where the environmental factors can strongly affect the content of secondary metabolites^35^.

The obtained results were in line with those reported previously, as thymol was always the main component, and the highest amount (91.15%) has been detected with *T. daenensis* from the Kurdistan region of Iran^14^. Due to the high content of thymol, *T. daenensis* can be considered as the main source of this valuable compound, which is the phenolic compounds with remarkable antimicrobial properties^36–38^.

Differently,^34,39,40^, reported that oxygenated sesquiterpene (35.3% and 33.14%) and oxygenated monoterpene (49%) were the main components of this essential oil obtained from plants collected in Ghamshelo and Arak, Iran. Hence, the high content of acids is a unique characteristic of the plants harvested from Isfahan province of Iran and can be strongly affected by the location, climatic and ecological conditions, field operations, growth stage, and genetic traits^41^. Thanks to its acid content, the essential oil from *N. sessilifolia* can exert several beneficial activities. Indeed, oleic Acid (9-Octadecenoic acid) is a component of omega-9 fatty acids, capable of counteracting cancer and cardiovascular diseases, autoimmune diseases, Parkinson’s and Alzheimer’s diseases, inflammatory diseases and hypertension^42–44^. Linoleic acid is one of the most unsaturated fatty acids of the human diet except for omega-6 fatty acids and has an active role in human growth and general health^42^.

This result is not completely in agreement with those of^24^ because only some compounds were similar but the most were different and none of the major constituents found in this essential oil was recorded by^24^. These results confirmed that environmental and climate conditions have a significant impact on the chemotypic properties of the obtained oil^45^. Caryophyllene oxide, which is contained in high amount in the oil from *H. incanus* collected in the Isfahan province of Iran, can inhibit the abnormal accumulation of fluid in the intercellular space of body tissues and it has been used as antitumor agent^46^.

In none of the previous studies, oleic acid has been reported as main component of the essential oil obtained from *S. inflata*, while palmitic acid (9.1%) has been indicated as the most abundant bioactive of the essential oil obtained from this plant by^28^. Germacrene D and bicyclogermacrene were found by other authors but in different amount: 8.9% and 5.1%^28^, 16.9% and 16.6%^29^, and 32.9% and 7.3%^30^. The differences found were mainly related to genetic or non-genetic variations connected with environmental differences such as soil chemical composition and physiographic factors.

These results perfectly fit with those reported by^18^, which found the same diameter of inhibition halo. The effective inhibition of the growth of *S. aureus* provided by this oil can be related to thymol content, which has antimicrobial properties. It is a phenol present in different essential oils with antibacterial activity thanks to its ability to improve the permeability of the membrane of the bacteria^38,47^.

All the other essential oils were not able to inhibit the growth of this fungal strain. Again, the antifungal activity of oil from *T. daenensis* may be related to the thymol, as it has good antifungal activity against a wide range of plant pathogenic fungi and food contaminants^36,37^. The mechanism of action of thymol has not been fully elucidated, but it is believed that it can damage the cell wall of the fungi or cause their cell wall to decay^48^.

According to these results,^18^ in their study, used the essential oil from the same plant (Daran, East of Esfahan province, Iran) and reported an effective inhibition of the growth of these two fungi while^49^, using the same oil, detected a good inhibition of *C. albicans* growth. Unfortunately, any essential oil obtained from the other species was capable of inhibiting the growth of these two fungal microorganisms.

To the best of our knowledge any activity against these two bacterial strains have been detected in previous studies. Further, the growth of *B. subtilis* and *P. vulgaris* was inhibited as well, even if in a lesser extent (14 ± 1 mm) than rifampin (19 ± 0 and 8 ± 0 mm) and gentamicin (30 ± 0 and 24 ± 0 mm) while^18^ found a remarkable effect of this oil against *B. subtilis* (43 ± 0 mm).

The efficacy of oil from *T. daenensis* against *E. coli* was confirmed by other researcher, even if the results were quite different and the diameter of inhibition halo varied from 7 to 44 mm^49,50^.

Differently,^23^ found a good activity against *N. asterotricha*, probably due to the presence of oleic acid, stearic acid, and linoleic acid, that have inhibitory activities against *S. aureus* and other microorganisms^51–53^. According to this, it has been previously reported that *P. aeruginosa* is highly sensitive to essential oils^54^.

*Candida albicans* is one of the most common pathogenic fungi capable of causing human infectious, which are often difficult to be threated because of the abuse of antibiotic occurred in the last decades^55^. The oil from *T. daenensis* represents a natural, promising alternative for the treatment of these infections and thymol seems to be the main responsible of its efficacy since its ability to penetrate the cell membrane and contribute to the clotting of cell contents^56^. The MFC of this oil against *C. albicans* (20 µg/mL) was also low and confirmed its effectiveness as antifungal agent^49^.

The strongest activity was found against the Gram-negative *P. aeruginosa* (MIC 63 μg/mL)*,* according to the results obtained by other authors against *H. calycinus*, *H. sessilifolius*. *Sh. dysenteriae, K. pneumonia*, and *S. paratyphi-A,* even if rifampin was significantly more effective (MIC 16 μg/mL)^57,58^.

In previous studies any antimicrobial effect was detected using the same oil obtained from plants collected from Isfahan Province, Iran^32^. These results confirmed the influence of plant habitat and conditions on the composition and activity of the essential oils. The MBCs/MFCs of the essential oil from *S. inflata* were always higher than MICs, indicating that the ability to inhibit the growth of bacteria was higher than that of killing them.

## Conclusion

In this study, for the first time, the essential oils obtained from *T. daenensis*, *N. sessilifolia*, *H. incanus*, and *S. inflata* growing in the Daran region of Isfahan (Iran), were obtained and their composition and antimicrobial activity were evaluated. The main common components were thymol, oleic acid, (−)-caryophyllene oxide, α-pinene, 1,8-cineole, palmitic acid, ( +)spathulenol, germacrene D, bicyclogermacrene, phytol, camphor, and borneol, 1,8-cineole and oleic acid, while others were randomly present as a function of the used plants. Essential oil from *T. daenensis* was the most active as it was able to inhibit the growth of different microorganisms, especially *S. aureus* and *A. brasiliensis*. Based on the MICs, the essential oils had low MICs and MBCs/MFCs and good effect on *Sh. dysenteriae*, *P. aeruginosa*, *E. coli*, *K. pneumonia* and *C. albicans*, then they can be used as natural and valid agents in agriculture, food, pharmaceutical and cosmetic industries for the treatment of microbial and fungal infections or contaminations.

## Data Availability

The datasets generated and/or analysed during the current study are available from the corresponding author on reasonable request.
